# Differentiation of human adipose-derived stem cells into cardiomyocyte-like cells in fibrin scaffold by a histone deacetylase inhibitor

**DOI:** 10.1186/s12938-017-0423-y

**Published:** 2017-11-23

**Authors:** Zahra Bagheri-Hosseinabadi, Parvin Salehinejad, Seyed Alireza Mesbah-Namin

**Affiliations:** 1 Department of Clinical Biochemistry, Faculty of Medical Sciences, Tarbiat Modares University, Tehran, Iran; 2 Department of Anatomy, Afzalipour School of Medicine, Kerman University of Medical Sciences, Kerman, Iran; 3 Physiology Research Center, Institute of Neuropharmacology, Kerman University of Medical Sciences, Kerman, Iran

**Keywords:** Fibrin scaffold, Differentiation, Human ADSCs, Cardiomyocyte**-**like cells, TSA

## Abstract

**Background:**

Human adipose-derived stem cells (hADSCs) are capable of differentiating into many cells such as cardiac cells. Different types of inducers are used for cardiac cell differentiation, but this question still remains to be investigated, which one is the best. The aim of this paper was to investigate the effect of combination of fibrin scaffold and trichostatin A (TSA), for differentiation of hADSCs into cardiomyocyte-like cells.

**Methods:**

After approval of characteristics of hADSCs and fibrin scaffold, hADSCs were cultured in fibrin scaffold with 10 µM TSA for 72 h and kept in standard conditions for 4 weeks. QRT-PCR and immunostaining assay were performed for evaluating the expression pattern of special cardiac genes and proteins.

**Results:**

In particular, our study showed that fibrin scaffold alongside TSA enhanced expression of the selected genes and proteins.

**Conclusions:**

We concluded that the TSA alone or with fibrin scaffold can lead to the generation of cardiac like cells in a short period of time.

## Background

Despite the advances made in scientific research and medical treatment of cardiovascular diseases, there are still patients who die from these diseases every year. Regenerative medicine with the use of autologous or allogeneic stem cells isolated from different tissues [1] is trying to repair damaged heart. Stem cells isolated from adipose tissue [2] with high number of pluripotent stromal cells are similar to bone marrow mesenchymal stem cells (BMSCs) in characteristics, surface proteins and differentiation potential [3, 4]. These cells exert their functions via their paracrine effect which improves angiogenesis, reduce ischemic-induced apoptosis, modulate inflammation and enhance progenitor cell recruitment and differentiation [5]. There is a lot of evidence regarding the ability of ADSCs to differentiate into different types of cells [6–8].

Culture on scaffolds is recently used for cell culture and stem cell differentiation. The morphology of the cultured cells in the scaffold is more similar to in vivo condition and the interactions, biology and functions of these cells are more natural like [9]. Amongst various scaffolds, fibrin scaffold is a natural biopolymer which could be prepared from plasma. Moreover, it is a biocompatible and easily polymerized polymer capable of fusing cells and active biological molecules. Fibrin could provide the conditions for direct the cell phenotype and differentiation and could act as scaffold for tissue engineering [10].

5-azacytidine (5-Aza) was one of the first inducers widely used to differentiate BMSCs into cardiomyocytes [11]. However, despite successful rabbit or mouse ADSCs differentiation, 5-Aza failed to trigger human ADSCs differentiation into contractile cardiomyocyte-like cells [12–14]. These deficiencies harbored by 5-Aza have lead researchers to use alternative agents like histone deacetylase inhibitor (HDAC inhibitors) such as trichostatin A. TSA is an organic compound that at first was used as an antifungal but later, its anticancer properties were discovered. It specifically inhibits the histone deacetylase families (HDAC) [15, 16]. Human ADSCs treated by TSA indicate more expression of cardiac genes [16].

Although the ADSCs have been shown to have the potential for differentiation into cardiomyocytes and vascular cells, a highly efficient differentiation protocol is yet to be reported [17, 18]. Therefore, in this study, we investigated this issue and found that TSA with scaffold is able to increase differentiation of hADSCs into cardiocyte.

## Methods

### Cell isolation

All materials were prepared from Sigma Company (Sigma-Aldrich, St. Louis, MO, USA) unless stated otherwise. Ethical approval was obtained from the Institutional ethical review board (approval number; IR. KMU. REC. 1394. 639) at Kerman University of Medical Sciences, Kerman, Iran. Human adipose–derived stem cells (hADSCs) were isolated from three 45 ± 3 old years women after a written consent had been obtained, using a standard cell culture protocol as previously described (article in review). Briefly, for primary culture of hADSCs, the adipose tissues were sliced into approximately 4 mm pieces and after washing with phosphate buffer saline (PBS) containing antibiotics, they were incubated with 0.5 mg/ml collagenase type IV, then centrifuged and the supernatant was removed and the cell pellet was suspended in culture medium containing; DMEM/F12 supplemented with 5% (v/v) FBS penicillin (100 U/ml), streptomycin (100 µg/ml), and amphotericin-B (2.5 µg/ml) and cultured on plate at 37 °C and 5% CO_2_. After 24 h, the medium was changed with fresh medium. The medium was replaced every 3 days until the cells reached an approximately 80% confluence.

### HADSCs properties

#### Phenotypic characteristics by flow cytometry

1 × 10^5^ cells/ml were fixed and incubated for 15 min at 4 °C with a 1:9 dilution of normal goat serum in PBS. The cells were then labeled with the following antibodies: FITC-conjugated anti-CD44, FITC-conjugated anti-CD34 (Chemicon; Temecula, CA, USA), FITC-conjugated anti-CD45 (Ediscience; USA), PE-conjugated-anti-CD73 (BD; San Jose, CA, USA), PE-conjugated anti-CD90 (Dako, Glostrup, Denmark) and PE-conjugated anti-CD105 (R&D; Minneapolis, MN, USA) for 1 h. The cells were washed and analyzed using a FACS Calibur (BD, NJ) machine and WinMDI software (BD Biosciences).

#### Induction of adipogenic, osteogenic and chondrogenic differentiation

To assess adipogenic differentiation, 1 × 10^3^ cells/cm^2^ were cultured in adipogenic differentiation medium (DMEM/F12 supplemented with 10% FBS, 100 nM dexamethasone, and 200 nM indomethacin). After 21 days, as accumulation of lipid-rich vacuoles is detected within cells, Oil Red O staining was done. Osteogenic differentiation was induced in osteogenic differentiation medium (50 µg/ml ascorbic acid, 10 nM dexamethasone, and 10 mM -glycerophosphate) with 3–5 × 10^3^ cells/cm^2^. After 21 days, Alizarin Red S staining, as determination of calcium accumulation assessment, was performed. Chondrogenic differentiation was persuaded by chondrogenic differentiation medium (Invitrogen, USA) in density of 3–5 × 10^3^ cells/cm^2^. After 21 days, chondrogenesis was evaluated using Alcian blue staining. The slides were fixed with 4% paraformaldehyde for 30 min and washed with PBS.

### Fibrin scaffold

#### Fabrication of fibrin scaffold and cell encapsulation

For preparation of fibrinogen solution, fresh frozen plasma (FFP) was obtained from Iranian Blood Transfusion Organization. FFP was thawed in water bath at 37 °C and then 10 ml of it was mixed with 10 ml of Protamine sulfate. The obtained solution was centrifuged at 1800*g* for 10 min. The supernatant was collected and 0.1 M Sodium citrate was added to the tube. Then, the fibrinogen solution was filtered by a 0.2 µm filter and prepared for usage as cell culture. Mean final fibrinogen concentration was 60 mg/ml. For encapsulation of the hADSCs into the fibrin gel, 200 µL of fibrinogen solution was added to a 5 cc syringe containing 1 × 10^5^ cells in 50 µl CaCl_2_ (50 mM) and 50 µl thrombin solution (Sigma-T6884, Thrombin from human plasma) (40 IU/ml), and allowed to polymerize for 10 min at 37 °C, CO_2_ incubator. Thus, hADSCs can be embedded in 3D fibrin gels. When the polymerization has ended and solution became gel, it was carefully immersed in a single well of a 6-well plates containing 2000 µl DMEM/F12 supplemented with 10% FBS, 1% antibiotic solution and 200 µl tranexamic acid, as an anti-fibrinolytic agent, then incubated at 37 °C in a humidified atmosphere containing 5% CO_2_ for different time points of growth after initial seeding.

#### Scanning electron microscopy (SEM)

The scaffold was washed with PBS and fixed with 2.5% glutaraldehyde at room temperature. After rinsing the scaffold with PBS and then with double deionized water (DDI), the fibrin scaffold was immersed in liquid nitrogen and kept in a freezer dryer (Super Modulyo, Edwards) [19]. The samples were coated with gold for 180 s by a sputter coater (SC7620, Emitech, UK) and observed under Scanning Electron Microscope (SEM, AIS2100, Seron technology, South Korea) at an accelerating voltage of 20 kV. The diameter of the fibers was calculated from SEM images by image analysis software (Image J, NIH, USA).

### Cell cycle analysis

Cell cycle analysis was performed by PI staining. In the first step, the cells from the fibrin scaffold and plate cell culture were detached at the 7th day after initial seeding. Then 1 × 10^6^ cells were put in a 1.7 ml tube and a solution containing 0.25 g sodium citrate, 0.005 g ribonuclease A, 0.025 g PI, and 0.75 ml Triton X-100 was added in 250 ml distilled water and incubated at 4 °C for 30 min. The cells were evaluated by FACS, and percentage of the cells in each phase of the cell cycle (G0/G1, S, and G2/M phases) was calculated using the FlowJo software, version 7.5 (Tree Star, Inc., San Carlos, CA).

### Induction of cardiac differentiation

In 2D group, 1 × 10^4^ cells/cm^2^ were seeded in cardiogenic medium, containing DMEM/F12 supplemented with 100 IU/ml penicillin, 50 µg/ml streptomycin, 5 µl of 0.25 mg/ml amphotericin and 10 µM TSA was added to the cells. In 3D group, the fibrin scaffold was incubated at standard conditions in 6-well plate for 7 days and then 2000 µl cardiogenic medium and 200 µl tranexamic acid were added into a single well and incubated in incubator at 37 °C in a humidified atmosphere containing 5% CO_2_. For both groups after 72 h, the medium was replaced by DMEM/F12 supplemented with 10% FBS and kept for 1, 2, 3, 4 weeks. To check for cell growth and morphological changes in 2D group, they were observed daily by inverted microscope, but the opacity of the scaffold did not allow the viewing of encapsulated cell in 3D by this type of microscope.

### Analysis of cardiogenic gene expressions by qRT-PCR

At weeks 1, 2, 3 and 4 of induction total RNA was extracted from treated and untreated hADSCs samples in 2D and 3D groups. RNA extraction was performed by precipitation method (RIBO-Prep, ILS) according to the manufacturer’s Instructions. After checking RNA integrity by denaturing agarose gel electrophoresis and ethidium bromide staining, cDNA was synthesized by Revert Aid (Thermo Scientific, USA). Quantitative PCR reactions were performed in duplicate on each sample of cDNA.

Aforementioned reactions were accomplished Maxima SYBR Green qPCR Master Mix (Fermentase, USA) in the presence of forward and reverse primers. SYBR green PCR amplifications were initiated at 95 °C for 10 min followed by 35 cycles of 95 °C, 15 s for denaturation; and 60 °C, 30 s for Annealing/extension. We aimed to analyze quantitative PCR on each sample by Rotor Gene 6000 machine (Qiagen, UK) for *NKX2.5*, *Cx43*, *cTnI*, *GATA4*, *HAND1*, *HAND2*, *βMHC* and *MLC2v* genes. Levels of each target gene expression were normalized to the expression of GAPDH (as an internal control) and calculated by the ΔΔCt method.

### Analysis of NKX2.5 and cTnI by immunocytochemistry

In the 2D group, the induced cells were fixed with 4% paraformaldehyde while in the 3D group, the scaffolds were fixed in 10% neutral buffered formalin for 12 h at room temperature and paraffin block and then sectioned (thickness 5 µm). Then, the fixed cells in 2 groups were incubated with 0.5% Triton X-100 containing PBS and 10% goat serum for 45 min at room temperature. The protocol was followed by a final incubation with *NKX2.5* (1:100; Biorbyte, UK) and *cTnI* (1:100; Abcam, USA) at 4 °C for 24 h. The next day, the cells were washed and incubated with goat anti-mouse IgG (ab150113, Abcam) and goat anti-rabbit IgG (ab150077, Abcam) at room temperature for 45 min. Then, the cell nucleus was stained by 5 µg/ml of Hoescht 33258 for 10 min. The images were taken under a fluorescence-inverted microscope equipped with a digital camera (DP71, Olympus, Japan).

### Statistical analysis

Data from the different tests are presented as mean ± SD of measurements in triplicate and were analyzed by T test and one-way ANOVA followed by post hoc Tukey’s test, after normality assumption. P < 0.05 was considered to be statistically significant.

## Results

### HADSCs properties

**Fig. 1:**
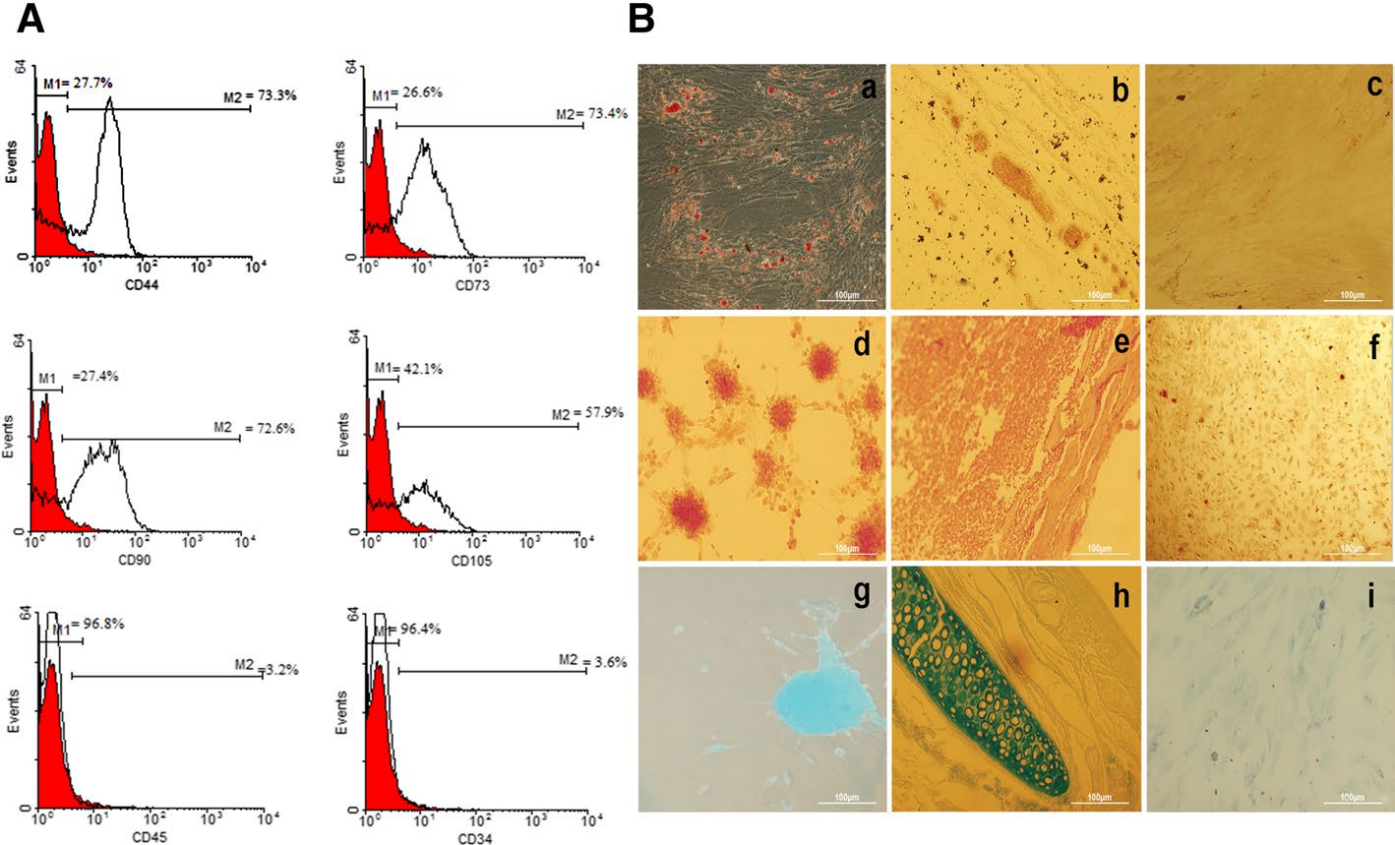
Flow cytometric analysis of surface-marker of human adipose derived stem cells (**A**). Differentiation of hADSCs into adipocytes, osteocytes and chondrocytes (**B**)

Flow cytometric analysis showed that the hADSCs were positive for mesenchymal cell markers; CD44, CD73, CD90 and CD105 and were negative for hematopoietic lineage markers; CD34, CD45 (Fig. 1A).

The presence of lipid droplets stained with Oil red O was observed in differentiated hADSCs (Fig. 1a). Alizarin red staining demonstrated calcium phosphate deposits in extracellular matrix of differentiated hADSCs (Fig. 1d). Chondrogenic differentiation of hADSCs was seen by Alcian blue staining (Fig. 1g). No spontaneous differentiation was observed in negative control cultures (Fig. 1c, f, i).

### Scanning electron microscopy (SEM)

**Fig. 2:**
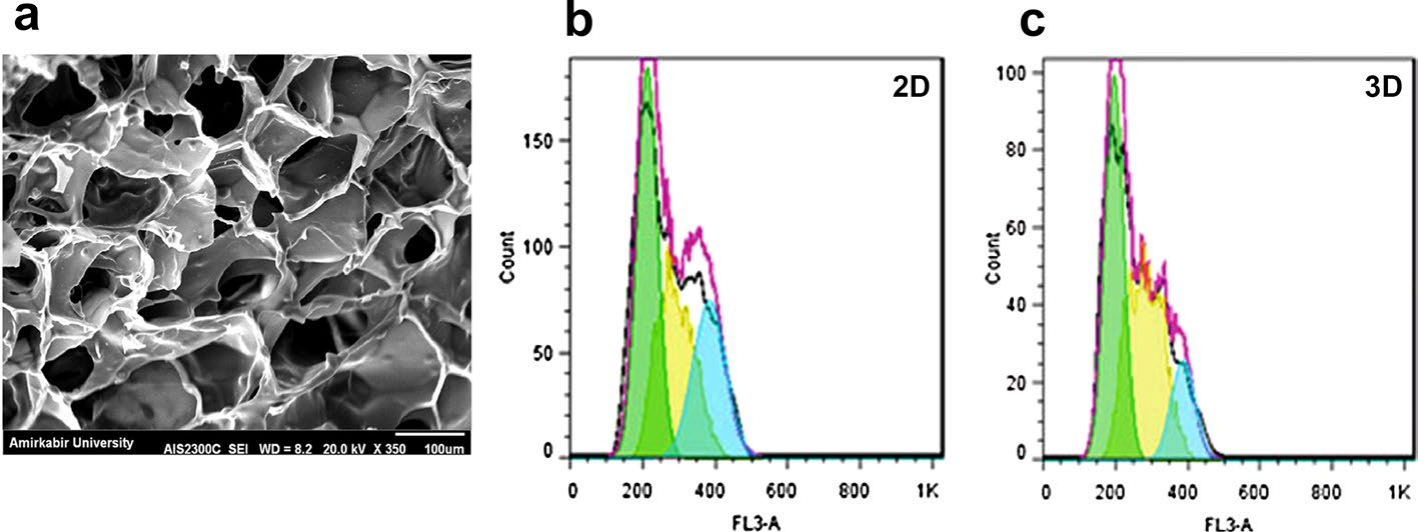
SEM image of fibrin scaffolds without cell (**A**). Distribution of hADSCs in different phases of cell cycle in 2D (**B**) and 3D (**C**) groups

Structure of the fibrin scaffold was appraised by SEM. A uniform structure of the scaffold with mean pore size of 113.14 ± 26 µm is shown in Fig. 2a.

### Cell cycle analysis

Seven days after initial culture, cell cycle analysis was done in two groups. Nearly 44.29% of 2D and 45.12% of 3D cells entered the G0/G1 phase. Moreover, 46.5% of 2D and 36.69% of 3D cells progressed into the S phase and 27.89 and 14.9% of the 2D and 3D hADSCs entered the G2/M phase, respectively (Fig. 2b).

### Cell morphology changes

**Fig. 3:**
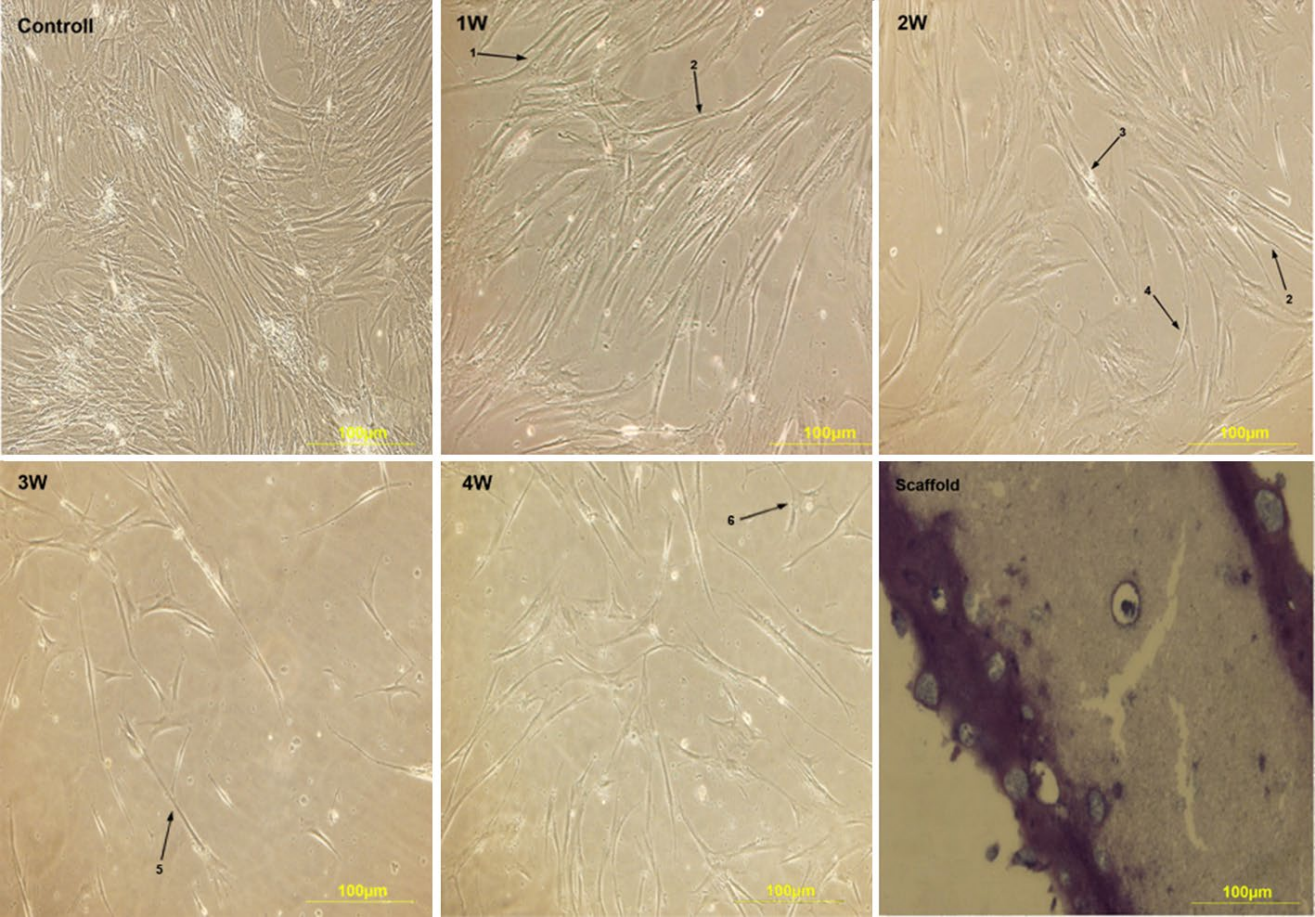
Morphological changes of differentiated hADSCs in 2D group during the first, second, third and fourth weeks. *1* multinuclear *2* elongated *3* ball-like *4* fork-like *5* myotube-like structure *6* star-like. Hematoxylin and eosin staining shows capsulated cells within scaffold pores in round and oval shaped

Morphological changes of the induced hADSCs with uninduced cells (negative control) during 4 weeks are compared in Fig. 3. The fibroblast-like morphology was the dominant form in negative control group. Gradual morphological changes of the induced hADSCs in the 2D group were multi nuclear, elongated, ball like, myotube-like structures and fork like, while they were star-like during the 4th week. Oval-like and round cell shapes were observed in margin and middle areas of fibrin scaffold. Despite the vast morphological changes among 2D cells, 3D cells almost maintained oval like form during all the weeks (Fig. 3).

### Analysis of cardiogenic genes by qRT-PCR

**Fig. 4:**
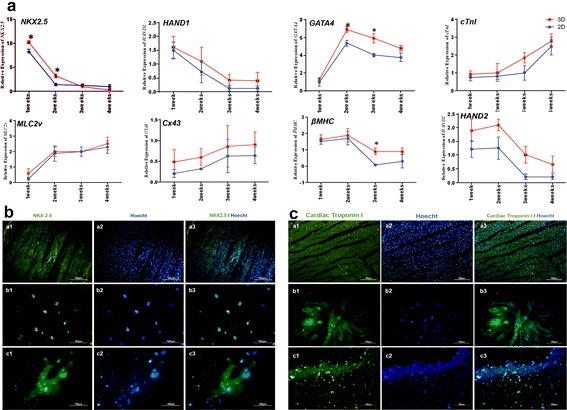
Expression of special cardiomyocyte genes by qRT-PCR in 2D and 3D groups (**A**). Significant increase of *NKX2.5* and *GATA4* and also significant decrease of *β-MHC* is observe in this figure (*P < 0.05; Data presented are mean ± SD. n = 3). Detection of *Nkx2.5* marker by immunocytochemistry (**B**) in 3D (*c*1, *c*2 and *c*3) and 2D (*b*1, *b*2 and *b*3) groups. Human heart stained as the positive control (*a*1, *a*2 and *a*3). Cell nuclei (blue) and *Nkx2.5* protein in the nucleus (green) stained by Hoechst. Detection of cardiac Troponin I marker by immunocytochemistry (**C**) in 3D (*c*1, *c*2 and *c*3) and 2D (*b*1, *b*2 and *b*3) groups. Human heart stained as the positive control (*a*1, *a*2 and *a*3). Cell nuclei (blue) and cardiac Troponin I protein in the nucleus (green) stained by Hoechst

In Table 1, final sequences of forward and reverse primers of desired genes are shown. QRT-PCR data are presented in Fig. 4a. Nearly all these genes had higher expression level in 3D group compared with 2D group. Also, differentiation was significantly overexpressed in some of the genes in 2 groups. *Cx43*, *cTnI*, and *MLC2v* had the same expression patterns with an upward trend from the first week to the 4th week in two groups. In contrast, *NKX2.5*, *HAND1*, H*AND2* and *β*-*MHC* had a downtrend from the first week to the 4th week in two groups. *GATA4* had different pattern from the other genes as it had a fast upward trend from the first week to the second week, then it had a slow downtrend from the second week to the 4th week. In addition, *NKX2.5* had statistically significant increase during the 1st and 2nd week while *GATA4* had the same trend as this increase in the 2nd and 3rd week in 3D group. Also, *β*-*MHC* gene showed a significant decrease in expression in 3rd week in 3D culture (Fig. 4a).

**Table 1:** Sequence specific primers for quantitative real-time PCR (qPCR)

Name	Forward sequence	Reverse sequence
Connexin43	5′ATGACCAGTCTGCCTTTCGTTG3′	5′CCATCAGTTTCGGCAACCTTG3′
GAPDH	5′TGCACCACCAACTGCTTAGC3′	5′GGCATGGACTGTGGTCATGAG3′
*cTnI*	5′CAAGCAGGTGAAGAAGGAGG3′	5′CTCAAACTTTTTCTTGCGGC3′
*MLC2v*	5′AAAGAGGCTCCAGGTCCAAT3′	5′CCTCTCTGCTTGTGTGGTCA3′
*GATA4*	5′GTGTCACCTCGCTTCTCCTT3′	5′GTGCCCTGTGCCATCTCT3′
*βMHC*	5′GATCACCAACAACCCCTACG3′	5′ATGCAGAGCTGCTCAAAGC3′
*NKX2.5*	5′GGTGGAGCTGGAGAAGACAG3′	5′AGATCTTGACCTGCGTGGAC3′
*HAND2*	5′TACCAGCTACATCGCCTACCT3′	5′TCACTGCTTGAGCTCCAGGG3′
*HAND1*	5′AGCCACCAGCTACATCGCCTAC3′	GCGATCCGCCTTCTTGAGTTC3′

### Immunochemistry analysis of cardiogenic protein

Immunochemistry staining of differentiated cardiomyocytes in 2D and 3D groups for *NKX2.5* (1st week) and *cTnI* (4th week) are presented in Fig. 4b, c, respectively. *Nkx2*.*5* was expressed in 39.66 ± 17 and 36.14 ± 12% (p = 0.16 and p = 0.20, respectively) of the cells in 3D and 2D cultures, respectively. In addition, *cTnI* was expressed in 26.33 ± 19 and 19.33 ± 7%, p = 0.11 and p = 0.16, respectively) of the cells in 3D and 2D groups, respectively. These differences between two groups were not significant (Fig. 4b).

## Discussion

The techniques for the induction of adult stem cells into cardiac cells need to be refined, since none of them couldn’t lead to the creation of functional cardiac cells [20]. Epigenetic modifications have attracted attention in recent years for producing cardiomyocytes from HDACs that have good potential for cardiac differentiation [21]. These epigenetic signatures such as methylation and acetylation on DNA bases or histone proteins have critical roles during the development and differentiation of cells [22]. Histone deacetylases (HDACs) are part of a vast family of enzymes that control cardiovascular growth, development and function [23]. HDACs adjust gene expression patterns by changing chromatin structure. Also, they deacetylate lysine residues and enforce compression of chromatin, decrease the access of transcription factors that are close to the promoters and finally cause gene silencing [24]. TSA, like most HDAC inhibitors, equally inhibits all known HDACs in a reversible fashion by displacing the requisite zinc ion within the active site [25] and removing genes from silent mode [24]. Also, in the process of cardiac cell differentiation, DNA methylation is important in preserving pluripotency and self-renewal of stem cells [17]. 5-Aza by inserting into DNA to form covalent bonds with DNA methyltransferase could inhibit methylation enzyme activity. It has been reported that this hypomethylation, after cell division, can reactivate transcription of genes previously silenced [7].

But, methylation levels from adipose tissue may be theoretically influenced by infiltrated cells, since adipose tissue samples are heterogeneous samples. Also, dynamic alterations in DNA methylation are related to the individual’s metabolic state such as; age, sex [26] obesity, caloric restriction, weight loss intervention, exercise, fat distribution and glucose homeostasis [27]. There is even an idea that human adipose mesenchymal cells are not methylated, so using 5-Aza for cardiac differentiation of adipose cells is accompanied with several limitations [17].

Although the cardiac-specific proteins can be expressed with mesenchymal stem cells (MSC) treated with 5-Aza, it seems that 5-Aza cannot be directly involved in stem cell differentiation. However, Wan Safwani et al. [28] believed that the effect of 5-Aza in cardiac differentiation may not be specific [28]. Consequently, effectiveness of 5-Aza should be experimentally determined for particular cells because this effectiveness depends on the hypomethylating level of the cells. Choi et al. [16] only those who compared 5-Aza with TSA on differentiation of hADSCs into cardiac cells. They failed to reproduce cardiac differentiation from hADSCs using 5-Aza until 3nd week while, TSA treatment increased expression level of some cardiac genes 11-fold in comparison with negative control in the 1nd week [16]. However, time is an important factor for cardiac differentiation. In addition, almost all of the studies that used TSA concluded that it was more effective than the other materials for cardiac differentiation [29–31].

In process of repair in the heart after an injury, in addition to need a suitable inducer, keeping the injected cells in the damaged area is important issue due to usually more than 94% of the transplanted cells leave the heart. So, using a compatible scaffold is the best way for overcoming this problem. Fibrin is a non-globular protein residing in plasma as fibrinogen precursors which can be polymerized by proteolytic activity of thrombin and makes a fibrin scaffold. If fibrin scaffold combined with mesenchymal cells can be completed the final outcome. Due to, fibrin scaffold have albumin, factor XIII, fibronectin and growth factors, especially platelet-derived growth factors that can be synthesized the matrix [32–34]. Also, within the fibrin network there is the arginine–glycine–aspartic acid (RGD) motifs that allows cell attachment, increased cell survival and binding of growth factors [32, 33]. This scaffold fabricated by fibrin proteins prepares an extracellular matrix like context in which embedded cells are able to simulate real ECM and tissue conditions [35].

Evaluation of the size of the scaffold pores is also optimal for each scaffold system. The pore sizes of the scaffold control its diffusion by transporting nutrients, removing waste, facilitating proliferation and migration of cells and determining the final mechanical properties of the scaffold [36, 37]. In this regard, the pore diameter of our scaffolds was 10.54 to 199.4 μm with an average size of 113.14 ± 26 μm.

HDAC inhibitors can affect gene expression patterns nearly in all cell types [38]. *NKX2.5* which is a key transcription factor and has a basic duty for heart formation was expressed at the highest level in 2D and 3D groups in the first week. Expression of *NKX2.5* represents immature cardiocytes. *HAND1* also had the same pattern. It is suggested that *HAND1* acts downstream of *NKX2.5* to control left ventricular development. At the next rank, *GATA4*, as early cardiac related gene marker, is expressed at its highest level in the second week. Cardiac contractile proteins (*cTnI*) and the gap junction protein (connexin43) are expressed at the highest level in the 4th week while another contractile protein, *βMHC* is expressed at the highest level in the second week. However, the amount of these three genes were not considerable. *HAND2* that was expressed with endocardium and epicardium mostly in the primitive right ventricular segment had its highest expression in the second week and after that had a downtrend that might be due to inappropriate and unspecific conditions of cultures.

In vivo studies have shown that there are several stages after an injury occurs to the heart. A blastema that includes cells and expresses early myocardial markers like *nkx2.5* and *hand2* is first formed. These markers are known as myocardial precursors and begin expression at 3–4 days after ventricular injury. In the second stage, developmental markers are induced and expanded by epicedial tissue and new epithelial coverage is created for the exposed myocardium. A subpopulation of epicardial cells undergoes epithelial-to-mesenchymal transition (EMT), makes inroads into the injury location and provides new vasculature to the regenerating muscle [39, 40]. Many signaling pathways promote cardiomyocyte proliferation, endocardium activation and epicardial EMT [40].

However, in the present study, *MLC2* *V* known as the structural gene in ventricular development had an expression pattern as *cTnI*. Overall, our results confirm that the first stage of cardiomyogenesis involves the formation of cardiomyoblasts, which express a clear subset of transcription factors, including *Nkx2.5* and *GATA4*. These genes are involved in activating the expression of the other specific genes to form the differentiated cardiomyocyte [41].

## Conclusions

Taken together, we show that TSA helps in fibrin scaffold stimulated differentiation of hADSCs towards cardiac cells during 4 weeks of the experiment. All of the genes were over expressed in the scaffold. This indicates that the fibrin scaffold by secretion of growth factor and other effective factors accompanying human adipose-derived stem cells have high potential for cardiac differentiation and could enhance the inductive abilities of TSA. However, to confirm this content, it is necessary for different dosages of TSA to be investigated in future.

## References

[1] Kastrup J (2011). Stem cells therapy for cardiovascular repair in ischemic heart disease: how to predict and secure optimal outcome. EPMA J.

[2] Mathiasen AB, Haack-Sørensen M, Kastrup J (2009). Mesenchymal stromal cells for cardiovascular repair: current status and future challenges. Future Cardiol.

[3] Chen HT, Lee MJ, Chen CH, Chuang SC, Chang LF, Ho ML (2012). Proliferation and differentiation potential of human adipose-derived mesenchymal stem cells isolated from elderly patients with osteoporotic fractures. J Cell Mol Med.

[4] Gronthos S, Franklin DM, Leddy HA, Robey PG, Storms RW, Gimble JM (2001). Surface protein characterization of human adipose tissue-derived stromal cells. J Cell Physiol.

[5] Panfilov IA, de Jong R, Takashima S-i, Duckers HJ (2013). Clinical study using adipose-derived mesenchymal-like stem cells in acute myocardial infarction and heart failure. Cell Cardiomyoplasts.

[6] Zuk PA, Zhu M, Mizuno H, Huang J, Futrell JW, Katz AJ (2001). Multilineage cells from human adipose tissue: implications for cell-based therapies. Tissue Eng.

[7] Huang JI, Zuk PA, Jones NF, Zhu M, Lorenz HP, Hedrick MH (2004). Chondrogenic potential of multipotential cells from human adipose tissue. Plast Reconstr Surg.

[8] Girao-Silva T, Bassaneze V, Campos LC, Barauna VG, Dallan LA, Krieger JE (2014). Short-term mechanical stretch fails to differentiate human adipose-derived stem cells into cardiovascular cell phenotypes. Biomed Eng Online.

[9] Liuyun J, Yubao L, Chengdong X (2009). Preparation and biological properties of a novel composite scaffold of nano-hydroxyapatite/chitosan/carboxymethyl cellulose for bone tissue engineering. J Biomed Sci.

[10] Janmey PA, Winer JP, Weisel JW (2009). Fibrin gels and their clinical and bioengineering applications. J R Soc Interface.

[11] Makino S, Fukuda K, Miyoshi S, Konishi F, Kodama H, Pan J (1999). Cardiomyocytes can be generated from marrow stromal cells in vitro. J Clin Invest.

[12] Lee W-CC, Sepulveda JL, Rubin JP, Marra KG (2009). Cardiomyogenic differentiation potential of human adipose precursor cells. Int J Cardiol.

[13] Strem BM, Zhu M, Alfonso Z, Daniels E, Schreiber R, Begyui R (2005). Expression of cardiomyocytic markers on adipose tissue-derived cells in a murine model of acute myocardial injury. Cytotherapy.

[14] Rangappa S, Fen C, Lee EH, Bongso A, Wei ESK (2003). Transformation of adult mesenchymal stem cells isolated from the fatty tissue into cardiomyocytes. Annals Thorac Surg.

[15] Oyama T, Nagai T, Wada H, Naito AT, Matsuura K, Iwanaga K (2007). Cardiac side population cells have a potential to migrate and differentiate into cardiomyocytes in vitro and in vivo. J Cell Biol.

[16] Choi YS, Dusting GJ, Stubbs S, Arunothayaraj S, Han XL, Collas P (2010). Differentiation of human adipose-derived stem cells into beating cardiomyocytes. J Cell Mol Med.

[17] Singla DK, Long X, Glass C, Singla RD, Yan B (2011). Induced pluripotent stem (iPS) cells repair and regenerate infarcted myocardium. Mol Pharm.

[18] Zhang J, Wilson GF, Soerens AG, Koonce CH, Yu J, Palecek SP (2009). Functional cardiomyocytes derived from human induced pluripotent stem cells. Circ Res.

[19] Lee JT, Chow KL (2012). SEM sample preparation for cells on 3D scaffolds by freeze-drying and HMDS. Scanning.

[20] Cheng K, Kuo T, Kul K, Hsiao C (2011). Human adipose-derived stem cells: isolation, characterization and current application in regeneration medicine. Genom Med Biomark Health Sci.

[21] Perea-Gil I, Prat-Vidal C, Bayes-Genis A (2015). In vivo experience with natural scaffolds for myocardial infarction: the times they are a-changin’. Stem Cell Res Ther.

[22] Gilardini Montani MS, Granato M, Santoni C, Del Porto P, Merendino N, D’Orazi G (2017). Histone deacetylase inhibitors VPA and TSA induce apoptosis and autophagy in pancreatic cancer cells. Cell Oncol (Dordr).

[23] Haberland M, Montgomery RL, Olson EN (2009). The many roles of histone deacetylases in development and physiology: implications for disease and therapy. Nat Rev Genet.

[24] Brougham CM, Levingstone TJ, Jockenhoevel S, Flanagan TC, O’Brien FJ (2015). Incorporation of fibrin into a collagen-glycosaminoglycan matrix results in a scaffold with improved mechanical properties and enhanced capacity to resist cell-mediated contraction. Acta Biomater.

[25] Lagace DC, Nachtigal MW (2004). Inhibition of histone deacetylase activity by valproic acid blocks adipogenesis. J Biol Chem.

[26] Keller M, Kralisch S, Rohde K, Schleinitz D, Dietrich A, Schon MR (2014). Global DNA methylation levels in human adipose tissue are related to fat distribution and glucose homeostasis. Diabetologia.

[27] Benton MC, Johnstone A, Eccles D, Harmon B, Hayes MT, Lea RA (2015). An analysis of DNA methylation in human adipose tissue reveals differential modification of obesity genes before and after gastric bypass and weight loss. Genome Biol.

[28] Wan Safwani WKZ, Makpol S, Sathapan S, Chua KH. 5-azacytidine is insufficient for cardiogenesis in human adipose-derived stem cells. J Negat Results BioMed 2012;11:3. 10.1186/1477-5751-11-3 PMC3274438 22221649

[29] Lim SY, Sivakumaran P, Crombie DE, Dusting GJ, Pebay A, Dilley RJ (2013). Trichostatin A enhances differentiation of human induced pluripotent stem cells to cardiogenic cells for cardiac tissue engineering. Stem Cells Transl Med.

[30] Yang G, Tian J, Feng C, Zhao LL, Liu Z, Zhu J (2012). Trichostatin a promotes cardiomyocyte differentiation of rat mesenchymal stem cells after 5-azacytidine induction or during coculture with neonatal cardiomyocytes via a mechanism independent of histone deacetylase inhibition. Cell Transplant.

[31] Lim SY, Sivakumaran P, Crombie DE, Dusting GJ, Pebay A, Dilley RJ (2016). Enhancing human cardiomyocyte differentiation from induced pluripotent stem cells with trichostatin A. Methods Mol Biol.

[32] Barsotti MC, Felice F, Balbarini A, Di Stefano R (2011). Fibrin as a scaffold for cardiac tissue engineering. Biotechnol Appl Biochem.

[33] Shachar M, Tsur-Gang O, Dvir T, Leor J, Cohen S (2011). The effect of immobilized RGD peptide in alginate scaffolds on cardiac tissue engineering. Acta Biomater.

[34] Ye Q, Zund G, Benedikt P, Jockenhoevel S, Hoerstrup SP, Sakyama S (2000). Fibrin gel as a three dimensional matrix in cardiovascular tissue engineering. Eur J Cardiothorac Surg.

[35] Discher DE, Mooney DJ, Zandstra PW (2009). Growth factors, matrices, and forces combine and control stem cells. Science.

[36] Lang NR, Munster S, Metzner C, Krauss P, Schurmann S, Lange J (2013). Estimating the 3D pore size distribution of biopolymer networks from directionally biased data. Biophys J.

[37] Loh QL, Choong C (2013). Three-dimensional scaffolds for tissue engineering applications: role of porosity and pore size. Tissue Eng Part B Rev.

[38] Glaser KB, Staver MJ, Waring JF, Stender J, Ulrich RG, Davidsen SK (2003). Gene expression profiling of multiple histone deacetylase (HDAC) inhibitors: defining a common gene set produced by HDAC inhibition in T24 and MDA carcinoma cell lines. Mol Cancer Ther.

[39] Curado S, Stainier DY (2006). The HeArt of regeneration. Cell.

[40] Missinato MA, Tobita K, Romano N, Carroll JA, Tsang M (2015). Extracellular component hyaluronic acid and its receptor Hmmr are required for epicardial EMT during heart regeneration. Cardiovasc Res.

[41] Karamboulas C, Swedani A, Ward C, Al-Madhoun AS, Wilton S, Boisvenue S (2006). HDAC activity regulates entry of mesoderm cells into the cardiac muscle lineage. J Cell Sci.

